# Strengths and weaknesses in the high-risk baby care network

**DOI:** 10.1590/1980-220X-REEUSP-2022-0150en

**Published:** 2023-03-13

**Authors:** Daniela Cardilli-Dias, Maristela Stoianov, Thaís Helena Ferreira Santos, Daniela Regina Molini-Avejonas

**Affiliations:** 1 Universidade de São Paulo, Faculdade de Medicina, Departamento de Fisioterapia, Fonoaudologia e Terapia Ocupacional, São Paulo, SP, Brazil.; 2 Universidade Anhembi Morumbi, São Paulo, SP, Brazil.

**Keywords:** Maternal and Child Healt, Child Healt, Health Service, Public Healt, Health Care Level, Child Development, Salud Materno-Infantil, Salud Infantil, Servicios de Salud, Salud Pública, Niveles de Atención de Salud, Desarrollo Infantil, Saúde Materno-Infantil, Saúde da Criança, Serviços de Saúde, Saúde Pública, Níveis de Atenção à saúde, Desenvolvimento infantil

## Abstract

**Objective:**

To characterize the Stork Network in health care for high-risk babies, pointing out its main challenges.

**Method:**

questionnaires were applied with those responsible for the babies and with network health professionals. Data were analyzed quantitatively.

**Results:**

statistically relevant variables were: link with the Basic Health Unit; individuals’ awareness of Family Health Support Center team; awareness of Family Health teams regarding the diagnosis of high-risk pregnancy and compliance with prenatal care; means of communication of individuals’ birth; awareness of the need for hospitalization as well as its duration; awareness of follow-up in Secondary Health Care; and its outcome, pointing to a difficulty in the axis of coordination and longitudinality of the services provided in the network.

**Conclusion:**

the greatest challenges lie in covering the territory by Family Health strategy teams, expanding teams and solidifying partnerships with Higher Education Institutions, guaranteeing a differentiated professional training.

## RESUMO

**Objetivo:**

caracterizar a Rede Cegonha na atenção à saúde ao bebê de alto risco, apontando seus principais desafios.

**Método:**

questionários foram aplicados com os responsáveis pelos bebês e com os profissionais de saúde da rede. Os dados foram analisados quantitativamente.

**Resultados:**

as variáveis estatisticamente relevantes foram: vínculo com a Unidade Básica de Saúde; conhecimento dos indivíduos sobre a equipe Núcleos de Apoio à Saúde da Família; ciência das equipes de Saúde da Família quanto ao diagnóstico de gravidez de risco e adesão ao pré-natal; via de comunicação do nascimento dos indivíduos; ciência quanto à necessidade de internação hospitalar, bem como sua duração; conhecimento sobre acompanhamento na Atenção Secundária à Saúde; e o desfecho do mesmo, apontando para uma dificuldade no eixo da coordenação e longitudinalidade dos serviços prestados na rede.

**Conclusão:**

os maiores desafios estão na cobertura do território pelas equipes de estratégia de Saúde da Família, na ampliação de equipes e na solidificação das parcerias com as Instituições de Ensino Superior, garantindo uma formação profissional diferenciada.

## RESUMEN

**Objetivo:**

caracterizar la Red Cigüeña en la atención a la salud de los bebés de alto riesgo, señalando sus principales desafíos.

**Método:**

se aplicaron cuestionarios con los responsables de los bebés y con los profesionales de salud de la red. Los datos se analizaron cuantitativamente.

**Resultados:**

las variables estadísticamente relevantes fueron: vinculación con la Unidad Básica de Salud; conocimiento de las personas sobre el equipo de los Centros de Apoyo a la Salud de la Familia; sensibilización de los equipos de Salud de la Familia sobre el diagnóstico del embarazo de alto riesgo y la adherencia al control prenatal; medio de comunicación del nacimiento de las personas; conciencia de la necesidad de hospitalización, así como de su duración; conocimientos sobre seguimiento en Atención Secundaria de Salud; y su resultado, apuntando a una dificultad en el eje de coordinación y longitudinalidad de los servicios prestados en la red.

**Conclusión:**

los mayores desafíos están en la cobertura del territorio por los equipos de la estrategia de Salud de la Familia, en la ampliación de los equipos y en la consolidación de alianzas con Instituciones de Educación Superior, garantizando una formación profesional diferenciada.

## INTRODUCTION

In the 1930s and 1940s, surveillance and education actions for women in the pre- and post-natal periods were intensified, called Maternity, Childhood and Adolescent Protection Programs, all submitted as proposals to the Brazilian National Department for Children (DNCr - *Departamento Nacional da Criança*)^(1,2)^.

In 2008, the Family Health Care Center (NASF - *Núcleo de Atenção à Saúde da Família*)^(3)^ emerged as a booster of comprehensive care, intervening in the culture of unnecessary referrals, in the articulation between levels of care, contributing to the discussion of professional training and encouraging reflection with managers on health indicators. The NASF aims to strengthen eight health care guidelines: interdisciplinarity, intersectoriality, territory, comprehensiveness, social control, Permanent Health Education, health promotion and humanization. These guidelines acted in eight strategic areas, such as physical activity/body practices, integrative and complementary practices, rehabilitation, food and nutrition, mental health, social work, child, adolescent and youth health, women’s health and pharmaceutical assistance.

NASF’s work stands out in the construction of a Unique Therapeutic Project for children with prematurity, malnutrition, developmental disorders, congenital and/or chronic diseases, among others. As responsible for promotion, prevention and rehabilitation, the NASF also monitors development up to the second year of life for groups of children and guardians, in addition to acting in training activities, such as training teachers and Community Health Workers (CHW), in identifying signs of risk for development. Considering that the NASF is an important Health Care Networks (RAS – *Redes de Atenção à Saúde*) articulator, being a member of primary care, organizing care, it has a special potential for acting in the Stork Network.

The Stork Network was implemented in 2011 by the Ministry of Health (MoH)^(4,5)^, characterized as a strategy by the Unified Health System (SUS – *Sistema Único de Saúde*), which guarantees all women the right to quality and safety in reproductive planning, humanized care during pregnancy, childbirth and the puerperium, guaranteeing children the right to attention and comprehensive care, from birth to 24 months of life, promoting healthy growth and development.

The reduction in infant mortality rates in Brazil denotes the importance of changes in health care directed to children, especially those directed to the perinatal period^(6)^. However, early and preventable neonatal deaths still occur. There is a need to improve and expand care services for health promotion and prevention of deaths in the first year of life in order to achieve infant mortality patterns similar to those of developed societies^(7)^.

Given the importance of articulating health actions in the prevention of injuries, health promotion or rehabilitation to improve the quality of maternal and child care^(8)^, this study aimed to characterize the Stork Network in health care for high-risk babies, pointing out its main challenges.

## METHODS

### Study Design

This is a quantitative cross-sectional study, carried out in two Basic Health Units (BHU), which act as points of articulation in the care of high-risk babies, which follow the mixed management model.

### Population, Location and Selection Criteria

The random choice was 22 children considered at high risk at birth, belonging to one of the equipment. The list with the list of these children was obtained through the Declaration of Live Births (DLB). As inclusion criteria, high-risk newborns were considered to be registered in health services according to the Ministry of Health criteria^(9)^, being at least 2 years old and at most 4 years old on the day of data collection, ensuring longitudinal use of the Care Network for high-risk babies with a focus on very early childhood, in addition to children born in the territory’s hospital. As exclusion criteria, non-consent from parents and/or guardians was considered.

Ten health professionals from the studied BHU participated. Inclusion criteria included composing the network responsible for the care of selected babies. Exclusion criteria included not consenting to participate in the research.

### Data Collection

It was carried out between 2016 and 2019, through a questionnaire prepared by the researchers, in two different versions: user group (UG) and professional group (PG), based on the Primary Care Assessment Tool (PCATool-*Brasil)* ^(10)^, only validated and standardized instrument for Portuguese to assess the Primary Health Care (PHC) guidelines in Brazil.

PCA-Tool adaptations aimed to characterize the Care Network in all its articulation points. The methodology for construction and validity of new instruments followed seven established steps: 1^st^ Establishment of conceptual structure; 2^nd^ Definition of objectives of the instrument and the population involved; 3^rd^ Construction of items and response scales; 4^th^ Item selection and organization; 5^th^ Instrument structuring; 6^th^ Content validity; and 7^th^ Pre-test^(11)^.

After completing all steps mentioned above, contact with the family was made via telephone or in person at the BHU. Contact with health professionals occurred through Family Health teams (FHt) responsible for the research subject. A meeting was scheduled between the researcher and the professionals who make up this team, to discuss the questionnaire application (PG). It is worth mentioning that families’ medical records were made available for reading and consultation of patients’ history. To characterize the sample, the 2015 Brazil Economic Classification Criteria socioeconomic questionnaire was applied^(12)^.

### Data Analysis

Data were registered and analyzed in a quantitative way, directed to the comparisons between the perceptions of UG and PG. In the analysis of descriptive statistics, qualitative variables were presented through their frequencies and percentages. As for inferential statistics for qualitative variables, Fisher’s exact test and the likelihood ratio were used to carry out comparisons of proportions between UG and PG. Agreement between groups was assessed using the Kappa statistic. Descriptive and inferential statistical analyzes were performed with SPSS, version 21 (SPSS 21.0 for Windows).

### Ethical Aspects

The article was accepted, under Opinion 2,079,844, approved in 2017. The research complies with Resolution 466, of December 12, 2012, and the Informed Consent Form was used as an instrument of consent.

## RESULTS

In sample characterization, the UG was composed of 22 subjects, and the PG, of 10 subjects, all were women. It was observed that, in the UG, 59.1% of subjects were up to 30 years old, and 40.9%, above that, while, in the PG, 30% of them were in the age group of up to 30 years, and 70%, above 30 years. In the head of household’s education variable, in the UG, 50% had completed high school and/or incomplete higher education, more than 22% had completed elementary school II/incomplete high school, 13.6% had completed elementary school I/incomplete elementary school II, and 13.6% completed higher education. In the PG, 90% of subjects indicate the head of household with complete higher education and only 10%, with elementary school II/incomplete high school. This variable also showed statistical significance.

Regarding the level of maternal education, the UG prevailed with 77.3% in complete elementary school II, high school and higher education, with only 22.7% illiterate, in addition to complete elementary school I and incomplete elementary school II. In the PG, in turn, 100% completed higher education. This variable had statistical significance.

Professional categories in the PG were medical staff, with 20%, and nursing staff, with 80%. As for their training time, it was found that 60% had graduated more than 11 years ago, and 40% less. Of the PG, 80% had a postgraduate degree and 20% did not. Half of PG subjects had worked in the SUS for more than five years, and the other half had worked for less than five. It was found that 80% of subjects worked on the equipment for more than a year, and only 20% for less than a year. Finally, in the UG, it was observed that more than 86% of subjects belonged to classes C, D, E, as opposed to PG, with 90% in classes A and B. This variable had statistical significance.

With regard to questions relating to the prenatal period (Table 1), approximately 55% of UG subjects stated that they had a relationship with a BHU professional, as opposed to more than 45% who stated that they had no relationship. In the PG, more than 60% of subjects pointed out the existence of a bond with a professional in health equipment; however, 13.6% denied it and almost 23% of these did not know how to answer (DNKA).

**Table 1.** Assessment of responses between the professional and user groups at the beginning of the prenatal flow/period – São Paulo, SP, Brazil, 2019.

Variables	User	Professional	p
**Is linked to BHU**	**N = 22**	**N = 22**	**0.004**
Yes	12 (54.5%)	14 (63.3%)	
No	10 (45.5%)	3 (13.6%)	
DNKA	0 (0.0%)	5 (22.7%)	
**Know NASF team**	**N = 22**	**N = 22**	**0.001**
No	19 (86.4%)	8 (36.4%)	
DNKA	0 (0.0%)	4 (18.2%)	
Yes/knows	1 (4.5%)	0 (0.0%)	
Yes/used	2 (9.1%)	10 (45.5%)	
**Professional who referred to RPN**	**N = 9**	**N = 10**	**0.014**
Nurse	5 (55.6%)	1 (10.0%)	
Doctor	4 (44.4%)	5 (50.0%)	
Doctor/nurse	0 (0.0%)	4 (40.0%)	

BHU – Basic Health Unit, NASF – Family Health Support Center, RPN – high-risk prenatal care.

It can be seen that 86.4% of UG subjects denied having awareness of the existence of NASF team at BHU, 4.5% reported being aware of it and only 9.1% claimed to have used the team. These data do not corroborate with PG responses, since 36.4% of families had no awareness of NASF, 18.2%, DNKA, and 45.5% of families used the NASF team in the territory.

The UG reported that 55.6% of subjects were referred to high-risk prenatal care (RPN) by the nursing team, and 44.4% by doctors, while PG reported that 10% of referrals were made by the nursing team, 40% shared nurse/doctor and 50% only by medical professionals.

In the neonatal period, it is noteworthy that 27.3% of PG could not mention anything about the occurrence of neonatal hospitalization as well as 76.5% of PG could not mention anything about the hospitalization ward. Still in the PG, contrary to what was found in the UG, 29.4% DNKA, 23.5% reported hospitalization for a period greater than 30 days, 35.3%, from 11 to 30 days, and 11.8%, up to 10 days. As for the means of communication about birth, 59.1% of UG declared that it occurred informally by the family, 27.3%, by counter-referral from the hospital, 9.1%, by active search of Family Health Strategy (FHS) and CHW and 4.5% DNKA. However, for the PG, 50% of subjects pointed out that birth was communicated by family/informally, 31.8% by FHS/CHW and 18.2% DNKA.

During the postnatal period (Table 2), it is notable that 63.6% of UG reported having been referred to Secondary Health Care (SHC), while 36.4% denied this. In the PG, half of subjects claimed to have been referred, 36.4% DNKA and 13.6% denied. Of the subjects who received a referral at hospital discharge, 92.9% of UG reported having received a referral and an appointment, while 7.1% reported having received only a referral. In the PG, 72.7% reported having received referral and scheduling and 27.3% DNKA. Thus, 100% of UG were referred to SHC later and, in the PG, half said yes and the other half DNKA.

**Table 2.** Assessment of responses between the professional and user groups at the end of the postnatal flow – São Paulo, SP, Brazil, 2019.

Variables	User	Professional	p
**Where puerperal follow-up took place**	**N = 22**	**N = 22**	**<0.001**
PHC	4 (18.2%)	15 (68.2%)	
SHC	2 (9.1%)	2 (9.1%)	
PHC/SHC	16 (72.7%)	4 (18.2%)	
DNKA	0 (0.0%)	1 (4.5%)	
**There was referral to SHC**	**N = 22**	**N = 22**	**<0.001**
Yes	14 (63.6%)	11 (50.0%)	
No	8 (36.4%)	3 (13.6%)	
DNKA	0 (0.0%)	8 (36.4%)	
**Referral mode**	**N = 14**	**N = 11**	**0.046**
Referral and scheduling	13 (92.9%)	8 (72.7%)	
Referral	1 (7.1%)	0 (0.0%)	
DNKA	0 (0.0%)	3 (27.3%)	
**Other referral to SHC**	**N = 8**	**N = 14**	**0.028**
Yes	8 (100%)	7 (50.0%)	
No	0 (0.0%)	0 (0.0%)	
DNKA	0 (0.0%)	7 (50.0%)	
**Duration of service**	**N = 22**	**N = 22**	**<0.001**
Up to 3 months	5 (22.7%)	1 (4.5%)	
> 3 months < 1 year	5 (22.7%)	2 (9.1%)	
> 1 year	12 (54.5%)	2 (9.1%)	
DNKA	0 (0.0%)	17 (77.3%)	
**Awareness of attendance or absence at SOC**	**N = 22**	**N = 22**	**0.001**
Yes	19 (86.4%)	11 (50.0%)	
No	3 (13.6%)	4 (18.2%)	
DNKA	0 (0.0%)	7 (31.8%)	
**Awareness mode of FHt**	**N = 19**	**N = 11**	**0.005**
PHC	3 (15.8%)	4 (36.4%)	
Family	16 (84.2%)	7 (63.6%)	
**Attendance at 1^st^ service at SOC**	**N = 22**	**N = 18**	**< 0.001**
Yes	22 (100.0%)	10 (56.6%)	
No	0 (0.0%)	4 (22.2%)	
DNKA	0 (0.0%)	4 (22.2%)	
**There was another absence**	**N = 22**	**N = 18**	**< 0.001**
Yes	12 (54.5%)	2 (11.1%)	
No	10 (45.5%)	0 (0.0%)	
DNKA	0 (0.0%)	16 (88.9%)	
**There was awareness by FHt for compliance**	**N = 22**	**N = 18**	**< 0.001**
Yes	12 (54.5%)	8 (44.5%)	
No	10 (45.5%)	4 (22.2%)	
DNKA	0 (0.0%)	6 (33.3)	
**There was rescheduling for SOC**	**N = 12**	**N = 2**	
Yes	7 (31.8%)	1 (50.0%)	**< 0.001**
No	3 (59.1%)	1 (50.0%)	
Abandonment	2 (9.1%)	0 (0.0%)	
DNKA	0 (0.0%)	0 (0.0%)	
**There was a search for other health equipment**	**N = 22**	**N = 18**	**< 0.001**
Yes	4 (18.2%)	4 (22.2%)	
No	18 (81.8%)	4 (22.2%)	
DNKA	0 (0.0%)	10 (55.6%)	

SHC – Secondary Health Care; SOC – Specialty Outpatient Clinics; FHt – Family Health team.

It can be seen that 54.5% of UG reported duration of care at Specialty Outpatient Clinics (SOC) for more than one year, 22.7%, between three months and one year as well as 22.7% also stated having held for less than three. Unlike UG, in PG, it was observed that 77.3% DNKA, while 9.1% scored the duration between three months and one year and, finally, 4.5% declared less than three months.

It was found that 86.4% of UG declared that the FHt was aware of their attendance or the absence of the first service at SOC, while 13.6% denied it. However, 50% of PG said yes, but 31.8% DNKA and still 18.2% denied awareness. As for the FHt’s awareness of attendance or lack of attendance at SOC, 84.2% of UG subjects stated that it was because of the family, and only 15.8%, because of PHC. In the PG, 63.6% of subjects stated that it was through family members, and 36.4%, through PHC. As for the questioning, there was a fact that there was presence/attendance at the first service at SOC, 100% of UG mentioned having attended, as opposed to the PG, in which 56.6% claimed to have attended, 22.2% denied and 22.2% DNKA.

In the UG, 54.5% had another absence, and 45.5% denied having being absent. It was observed that 88.9% of PG DNKA, and only 11.1% claimed to be absent. A total of 54.5% of UG pointed out that there was sensitization by FHt compliance with treatment in SHC, while 45.5% said no. In the PG, 44.5% said yes, 33.3% DNKA and 22.2% denied.

Finally, with respect to the Care Network (Table 3), it can be observed that most UG (45%) denied being aware of the existence of intrasectoral articulation between PHC and SHC, 31.8% stated being aware and 22.7% DNKA. In the PG, 40% of subjects denied their existence, but another 40% claimed to have awareness and even 20% of these DNKA. It can also be identified that 45% of UG denied having awareness of the existence of intrasectoral articulation between PHC and Tertiary Health Care (THC), still 50% DNKA and only 4.5% claimed to being aware of this information. On the other hand, 80% of PG denied the existence, in addition to 20% of those DNKA.

**Table 3.** Assessment of responses between the professional and user groups regarding the Care Network for high-risk babies – São Paulo, SP, Brazil, 2019.

Variables	User	Professional	p
**There is ISA between PHC and SHC**	**N = 22**	**N = 10**	**<0.001**
Yes	7 (31.8%)	4 (40.0%)	
No	10 (45.5%)	4 (40.0%)	
DNKA	5 (22.7%)	2 (20.0%)	
**There is need for ISA between PHC and SHC**	**N = 15**	**N = 6**	**0.004**
Yes	14 (93.3%)	6 (100.0%)	
No	0 (0.0%)	0 (0.0%)	
DNKA	1 (6.7%)	0 (0.0%)	
**ISA time between PHC and SHC**	**N = 7**	**N = 4**	
> 1 year	0 (0.0%)	3 (75.0%)	**<0.001**
DNKA	7 (100.0%)	1 (25.0%)	
**ISA between PHC and SHC is functional**	**N = 7**	**N = 4**	**<0.001**
Yes	7 (100.0%)	2 (50.0%)	
No	0 (0.0%)	2 (50.0%)	
DNKA	0 (0.0%)	0 (0.0%)	
**There is ISA between PHC and THC**	**N = 22**	**N = 10**	**<0.001**
Yes	1 (4.5%)	0 (0.0%)	
No	10 (45.5%)	8 (80.0%)	
DNKA	11 (50.0%)	2 (20.0%)	
**Knows the attributes of network professionals**	**N = 22**	**N = 10**	**<0.001**
Yes	8 (36.4%)	9 (90.0%)	
No	14 (63.6%)	1 (10.0%)	
DNKA	0 (0.0%)	0 (0.0%)	
**The care network is functional**	**N = 22**	**N = 10**	**<0.001**
Yes	18 (81.8%)	2 (20.0%)	
No	3 (13.6%)	5 (50.0%)	
DNKA	1 (4.5%)	3 (30.0%)	
**Network is of quality**	**N = 22**	**N = 10**	**<0.001**
Yes	18 (81.8%)	3 (30.0%)	
No	4 (18.2%)	4 (40.0%)	
DNKA	0 (0.0%)	3 (30.0%)	

ISA - intrasectoral articulation; PHC - Primary Health Care; SHC - Secondary Health Care; THC - Tertiary Health Care.

A total of 63.6% of UG reported not knowing the attributes of professionals belonging to the Care Network for high-risk babies and only 36.4% said they knew. In the PG, 90% of subjects recognized that they knew the attributes, and 10% denied. Regarding the Care Network functionality, 81.8% of UG stated that it was functional, 13.6% denied it and 4.5% DNKA. Half of PG subjects denied functionality, 30% DNKA and only 20% claimed to be functional. As for Care Network quality, 81.8% of UG declared it to be of quality, but 18.2% denied this fact. Moreover, 40% of PG subjects declared not to be qualified, 30% DNKA and only 30% of these recognized it as being of quality.

## DISCUSSION

The predominantly female role in the sample of this study is highlighted, as responsible for home, family and child care as well as in the conquest of a greater space in the professional scope. Women have been playing numerous roles imposed by the social context in which they find themselves^(12,13)^.

Still regarding sample characterization, important differences were observed between UG and PG, with emphasis on education and socioeconomic level, which may have influenced the answers to the questions asked. Studies claim that maternal education has an impact on child development and health^(14)^. Economic and social conditions directly influence individuals’ and populations’ health conditions. This context that briefly defines all the social, economic, political, cultural and environmental determinants of health is called “social determinants of health”, which feedback and benefit each other mutually^(15)^.

In this research, the beginning of the flow or prenatal period was called the set of questions applied about the events that occurred with subjects in health services during the entire prenatal period. Among these issues, it can be observed that only three variables showed a statistically significant difference, apparently indicating a divergence of responses between the two groups: having a link with BHU, knowing the NASF team and professionals who referred to RPN.

In PHC, the bond becomes elementary school for FHS, in order to increase compliance and success in disease treatments as well as the prevention of injuries for all families belonging to the territory^(16,17)^. The NASF team formation and implementation in the territory has as one of the many attributions to support the work carried out by FHS, in order to join efforts to expand the breadth and scope of primary care actions, as well as their resolvability, helping to implement health promotion, disease rehabilitation and disease prevention, building comprehensive care for SUS users^(18–21)^. Referral to RPN ensures a peculiar look at maternal and child health, as advocated by the Ministry of Health. Therefore, all professionals involved in the Care Network for high-risk babies, belonging to PHC, must pay attention to the presence or emergence of high-risk factors, and should also be trained to assess each dyad, in order to identify the need or not to be referred to RPN quickly and at any time during the gestational period. To this end, the identification of high risk for pregnant women and/or fetus must be carried out from the first consultation and must be reviewed at each return visit, i.e., continuously. The team must keep informed through the counter-referral of other health equipment and also through the active search of pregnant women in their territory of operation^(20–22)^.

After discussing the information about the beginning of care in the network, the middle of the flow or neonatal period and the set of questions applied about the events in health services during the entire neonatal period stand out. When comparing responses between PG and UG, few variables showed statistically significant differences, namely: there was hospitalization; inpatient ward; hospitalization time; communication about birth. Unfortunately, there is still the presence of devaluation of records in public and private institutions of health professionals. This fact can contribute to misconduct, impairing the evolution of cases or even favoring death. It is necessary, for the network quality, to use the record of all procedures and flows by the professionals involved. Therefore, awareness of prenatal care, childbirth and postpartum information, as well as its formal registration, done properly, becomes essential for an effective work by FHS. The moment of prenatal care is highly representative in premature labor prevention, such as the identification of high-risk factors, actions to prevent injuries, conventional and protocol care practices and health promotion actions^(19–23)^.

Finally, there are questions about the postnatal period. When comparing responses between UG and PG at the end of the postnatal flow/period, only some variables showed statistically significant differences, indicating divergence of responses between the two groups. It is noteworthy that there was a referral to SHC, another referral to SHC, awareness of attendance or absence at SOC, FHt awareness mode, attendance at the 1^st^ service at SOC, another absence and awareness by the FHt for compliance. The puerperal follow-up aims to assess the general state of mother-baby dyad’s health, guide the family on basic hygiene care, vaccination, breastfeeding, family planning, identify high-risk situations and/or intercurrences at the time of consultation or hospital discharge, and diagnose them and refer them to specific professionals and/or specialized equipment according to pre-established high-risk criteria^(20, 21, 22, 23, 24, 25)^. The NASF team will also be able to participate in this process as a support team for FHS so that it takes place in time and satisfactorily, in order to contribute to the broader perspective of different professional categories^(20–26)^.

The maintenance of records, whether physical or electronic, is essential for the longitudinal follow-up of subjects in question to occur fully and in a qualified manner. Longitudinal care is a competence of PHC and contributes to comprehensiveness and coordination of care. However, ensuring this practice still represents a major challenge for SUS^(22)^.

Finally, the questions addressed to subjects in question regarding their awareness and opinion regarding services provided by the network’s health equipment in their region were defined as being a Care Network for high-risk babies. When comparing responses between UG and PG, only some variables showed statistically significant differences, indicating divergence of responses between the two groups. Such divergence was observed for the variables: intrasectoral articulation (ISA) between PHC and SHC; ISA between PHC and THC; know the attributes of network professionals; functional network; and quality network.

Given the awareness of ISA, as in the Care Network itself, as exposed by both, the existence of the Brazilian National Health Promotion Policy (PNPS – *Política Nacional de Promoção da Saúde*) is highlighted. This presents a set of strategies and ways of producing health at the individual and collective levels, characterized by intra and intersectoral articulation and cooperation, through RAS formation. Its principles aim at the construction and articulation of cooperative and problem-solving networks, which collaborate for a comprehensive and equitable health practice, and such articulations are necessary^(27)^.

The network structuring implies willingness of each health service to review itself, to carry out a humanized work, to promote continuous updating of professionals of system teams as a whole, to guarantee the participation of local and regional managers, in addition to the population itself as the leading roles of their health, in order to enforce practice and, in this way, achieve a qualified and functional Care Network^(20–28)^.

It is known that the health network in the territory is made up of public facilities that are organized in a hierarchical and regionalized manner into three levels of care: basic, medium and high complexity. Primary care stands out as the coordinator of care in the territory, with health promotion actions, prevention of diseases and injuries, carrying out diagnosis, treatment, rehabilitation and health maintenance. In this scenario, the NASF were created to expand the scope of primary care actions, through the support of FHt in the network of services and in territorialization and regionalization processes^(29)^.

It is necessary to reflect on the performance as a member of an FHt so that it is effective as a network articulator, questioning: Are Higher Education Institutions contributing to health professionals’ education, qualified to compose the current job market in a more humanized and collective way, through interprofessional education (IPE)? It is necessary to be clear about its importance in the implications for the quality of care offered within SUS^(30)^.

The article contributes to the expansion of awareness in the field of care for children at high risk in the RAS in Brazil, bringing new and relevant information about the challenges encountered in the Stork Network. Brazilian public health policies, over the decades, have played an important role in empowering the self-care of subjects and their families as well as in the practice of health professionals involved in this context. Therefore, understanding how the Stork Network works, with its weaknesses and strengths, allows the identification of a range of possibilities for actions and interventions regarding care of high-risk babies.

The study carried out had limitations regarding sample size, which had a small number, possibly due to the difficulty of identifying subjects in the network, suggesting a look at the implementation of information systems that could contribute to an expansion and qualification in health equipment articulation within the Care Network for high-risk babies. Another issue concerns subjects’ availability of time to answer the instrument as well as to compose the research, interpreting the questionnaire as an evaluative means of the work carried out by them.

## CONCLUSIONS

Considering the above, it is believed that the greatest challenge is the total territory coverage by the FHt, with a reduction in the number of families assisted per team, in parallel with the expansion of NASF teams.

Emphasis is also placed on training and professional updating through permanent education for all actors involved in the process so that there is a consistent contribution to a more effective and qualified Care Network.

Therefore, new studies in this area, with a larger number of subjects, in different municipal, state or national territories, carried out longitudinally, may expand Brazilian scientific findings, as well as contribute to a comprehensive, humanitarian and equitable health practice, promoting maternal and child health in a holistic and effective way.

## Financial support

 This work was carried out with the support of the Coordination for the Improvement of Higher Education Personnel – Brazil (CAPES – *Coordenação de Aperfeiçoamento de Pessoal de Nível Superior*) – Financing Code 001.
